# Assessment of left ventricular systolic function by non-invasive pressure-strain loop area in young male strength athletes

**DOI:** 10.1186/s12947-020-00227-w

**Published:** 2020-11-13

**Authors:** Pengge Li, Yonggao Zhang, Lijin Li, Yingchun Chen, Zhen Li, Songyan Liu, Shaohua Hua

**Affiliations:** 1 Department of Ultrasound, the First Affiliated Hospital of Zhengzhou University, Zhengzhou, 450000 Henan China; 2 Department of Radiology, the First Affiliated Hospital of Zhengzhou University, Zhengzhou, 450000 Henan China; 3 GE Healthcare Ultrasound Clinic Education Team, Zhengzhou, 450000 China

**Keywords:** Echocardiography, Myocardial work, Athlete heart, Two-dimensional speckle tracking, Pressure-strain loop

## Abstract

**Background:**

The health of athletes has been recognized as a worldwide public concern with more reported sudden cardiac deaths (SCD). Therefore, early detection of abnormal heart function in athletes can help reduce the risk of exercise. A novel valid non-invasive method to evaluate left ventricular (LV) myocardial work (MW) using LV pressure-strain loop (PSL), was used in this paper to explore LV systolic function in young male strength athletes.

**Methods:**

Thirty-six professional young male strength athletes (the athlete group) and 32 healthy, age-matched young men (the control group) were involved in the study. The LVMW parameters were calculated as the area of PSL by two-dimensional speckle tracking echocardiography (2D-STE) and peak systolic LV pressure. The differences between two groups of data and the predictive efficacy of MW parameters for LV systolic function were analyzed.

**Results:**

The athlete group had significantly higher values of global wasted myocardial work (GWW) and peak strain dispersion (PSD) than did the control group (*P*<0.05). Global myocardial work index (GWI), global constructive myocardial work (GCW) and global longitudinal strain (GLS) were lower in the athlete group than that in the control group, although statistical significance was not reached (*P*>0.05). Due to the proportion of GWW and GCW, statistically significant reduction was found in global myocardial work efficiency (GWE) in the athlete group. Conventional echocardiography parameters were well correlated with GWW and GWE (*P*<0.05). The best predictor of LV myocardial contractile performance in the athletes using receiver operating characteristic curve (ROC) was GWE, with the area under ROC (AUC) of 0.733, sensitivity of 83.3% and specificity of 59.4%.

**Conclusions:**

Subclinical changes have appeared in the hearts of young male strength athletes after long-term intensive exercise and LVMW parameters by PSL play an important role in the evaluation of athlete’s LV contractile performance.

## Introduction

The concept of “athlete’s heart”, including physiological adaptation and pathological changes both in cardiac morphology and function after prolonged and intensive exercise, was firstly raised by the end of nineteenth century [1–3]. To date, a growing number of reports on sudden cardiac death (SCD) of athletes caused by long-standing practice has made it imperative to screen athletes for the prevention of SCD [4, 5]. With the wide application of echocardiography and the continuous development of new ultrasonic technology, studies on the athletes’ hearts are getting more thorough, making it play a pivotal role in detecting and monitoring the subclinical changes of athletes’ cardiac morphology and function in time [6].

As a new parameter taking both deformation and afterload of left ventricular (LV) into account, myocardial work (MW) potentially could provide more reliable value to myocardial function assessment than that of strain only [7]. Russell et al. [8] has demonstrated the effectiveness of the non-invasive method to evaluate LVMW, via combining LV strain data by two-dimensional speckle tracking echocardiography (2D-STE) with non-invasive estimated LV pressure curves from brachial artery and correlated well with invasive measurements [9]. The clinical values of this new approach have potential in predicting response to cardiac resynchronization therapy and assessing myocardial contractility in hypertension and dilated cardiomyopathy [10, 11].

The purpose of this study aimed to: (i) describe the basic echocardiography characteristics of LV in the hearts of the athletes and (ii) explore the possibility of LVMW parameters to evaluate myocardial function among the athletes.

## Methods

### Study population

The study was performed in 36 male athletes engaged in wresting (average age 19.50 ± 1.38 years) and 32 control individuals (average age 20.03 ± 1.06 years). The inclusion criteria of the athlete group were training years≥5, training time ≥ 30 h per week, never stop training and never use doping substances. The healthy controls with no records of participation in long-term intensive training were collected from the physical examination center of the First Affiliated Hospital of Zhengzhou University. Meanwhile, the participants having good image quality for myocardial speckle tracing analysis and without cardiovascular disease such as arrhythmia, valvular stenosis or regurgitation were necessary. Subjects that did not fulfill all the inclusion criteria were excluded. The study was authorized by the local ethics committee, and written informed consent was obtained.

### Echocardiographic analysis

Transthoracic echocardiography was performed to obtain images for analysis, employing a Vivid E95 ultrasound system equipped with a M5S 3.5mHz transducer (GE Vingmed Ultrasound, Horten, Norway). The participants maintained a left lateral decubitus position when scanning under peaceful breath and connecting the electrocardiogram synchronously. Collect standard 2D gray-scale dynamic images consisting of three consecutive cardiac cycles in long-axis, apical two-chamber and four-chamber views, then put in a certain workstation for offline analysis. Ultrasonic recordings and measurements were processed in line with the recommendation of the American Society of Echocardiography and European Association of Cardiovascular Imaging [12].

Left ventricular end-diastolic diameter (LVDd), diastolic interventricular septal thickness (IVSTd), and diastolic posterior wall thickness (PWTd) were obtained in the parasternal long-axis section of LV, and relative wall thickness (RWT) was computed using the ratio: (IVSTd+PWTd)/LVDd. Left ventricular mass (LVM) was obtained by the standard cube formula and normalized to body surface area (BSA). LV end-diastolic volume (EDV), end-systolic volume (ESV), stroke volume (SV) and cardiac output (CO) were measured and indexed to BSA using biplane Simpson’s method as recommended [13], and LV ejection fraction (EF) was calculated.

### Strain analysis by 2D-STE

2D gray-scale images were obtained from the apical two-chamber, four-chamber and long-axis views at frame rates ≥60 frames/sec. By manually clicking on the mitral annulus and apex of three sections at the end-systolic frame, the region of interest between the endocardium and epicardium was automatically defined using 2D-STE and manually adjusted if necessary [14]. Then, the assessment of global longitudinal strain (GLS) was acquired and peak strain dispersion (PSD) was subsequently determined. Aortic valve closure was automatically defined in the LV apical long-axis view [7]. The GLS of LV was calculated from the average value of the three views, including 17 segments of the myocardium.

### Myocardial work analysis

**Fig. 1:**
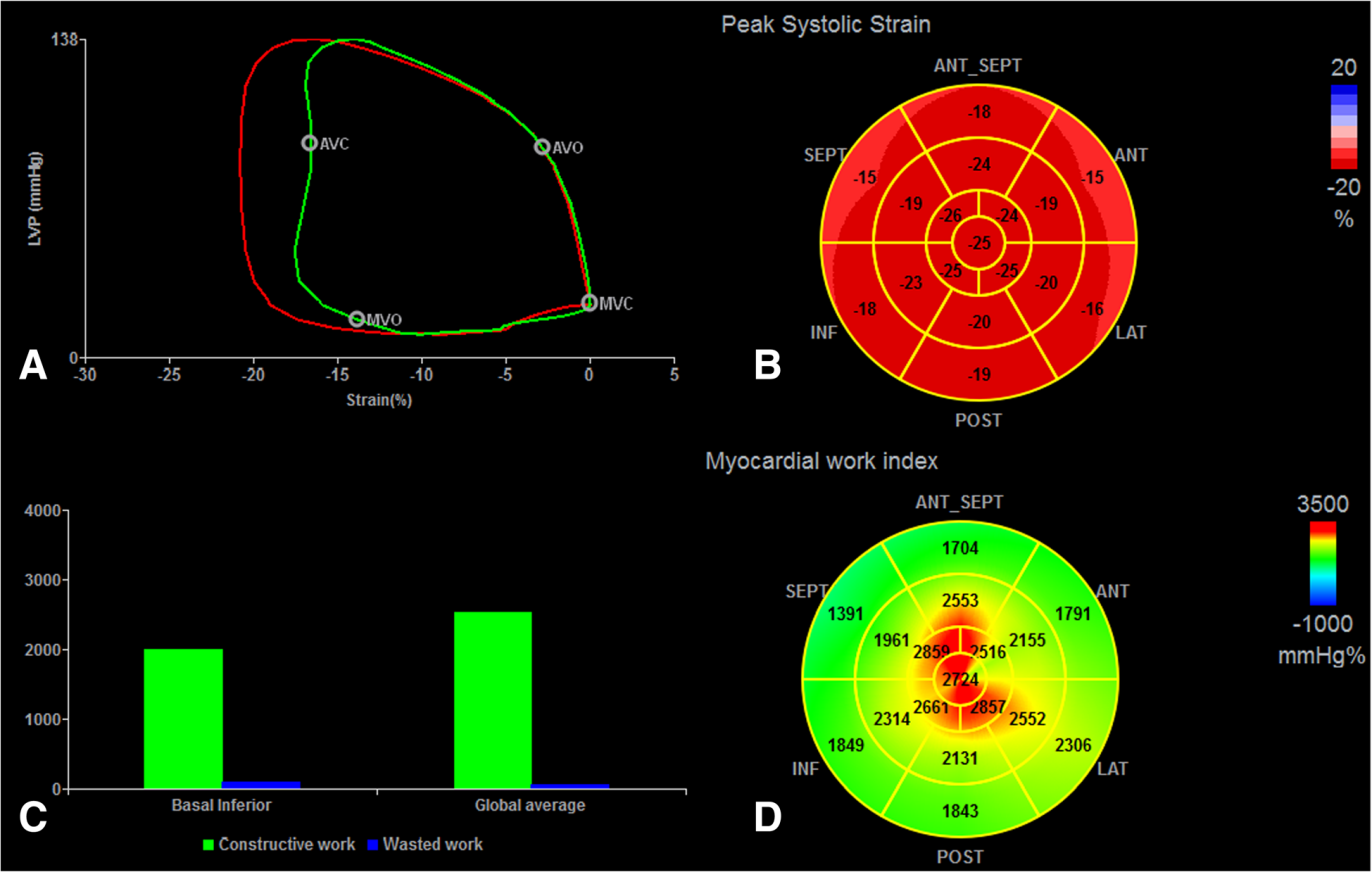
**a** Non-invasive LV PSL diagram of the athlete group. The red loop area represented the average LV global MW index and the green loop area represented MW of basal inferior. **b** 17-segment bull’s-eye representation of GLS in an athlete. **c** The MW of global average and basal inferior in an athlete with the green representing constructive work and the blue expressing wasted work. **d** 17-segment bull’s-eye expression of GWI with areas of normal in green and high in red. MVC, mitral valve closure; AVO, aortic valve open; AVC, aortic valve closure; MVO, mitral valve open

LVMW parameters were calculated from non-invasive pressure-strain loop (PSL) area as previously described (Fig. 1). Brachial cuff pressure measured before echocardiographic study was used to substitute for aortic pressure as peak systolic LV pressure. Then, the LV pressure curve was constructed by defining the isovolumetric and ejection time based on the valvular timing events via the software (EchoPAC ver. 202, GE Vingmed Ultrasound, Norway) [15]. Furthermore, the replicability of the non-invasive pressure curve was demonstrated in a dog model and in patients with diverse cardiac disorders [10].

**Fig. 2:**
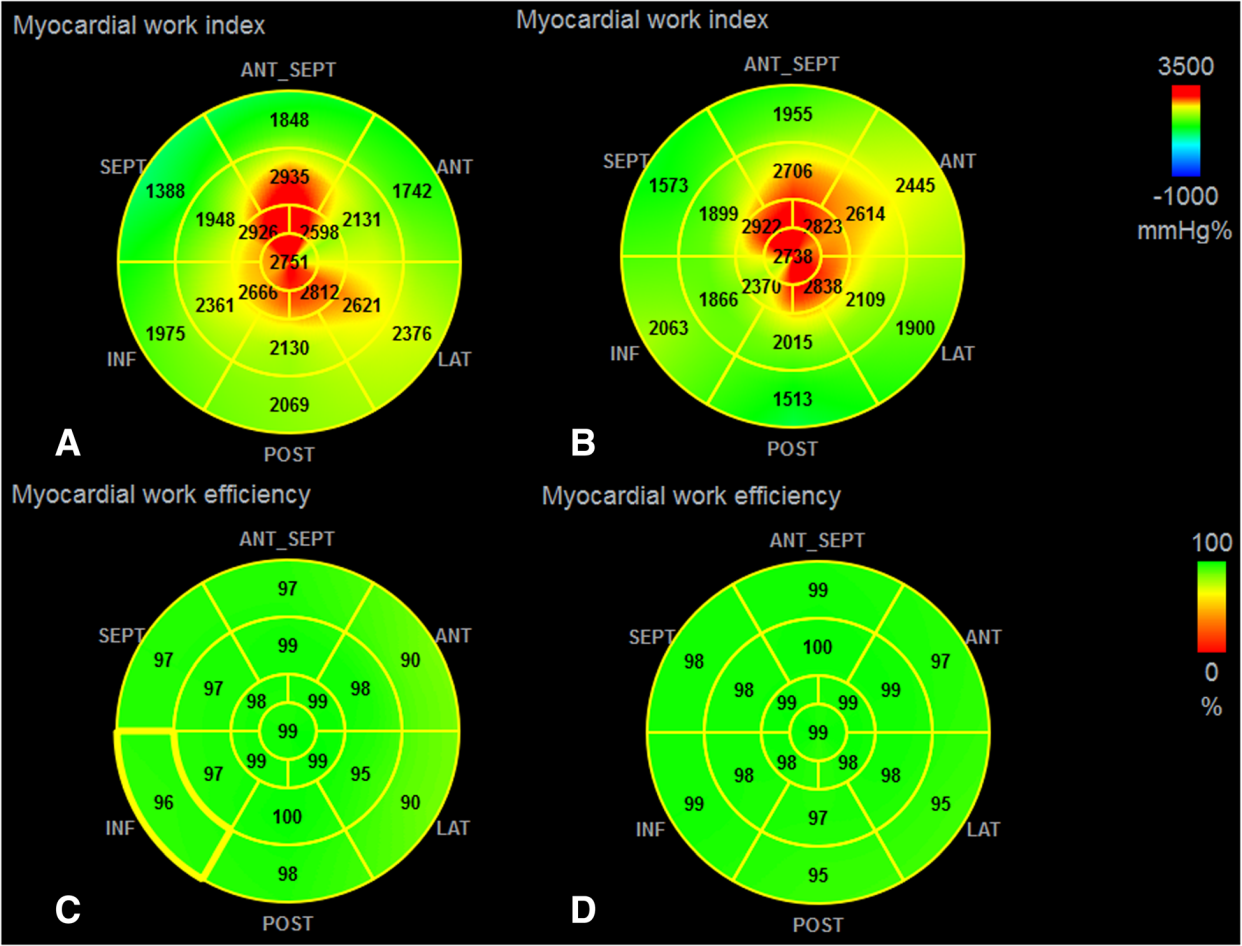
**a** and **c** 17-segment bull’s-eye representation of MW index and myocardial work efficiency from an athlete (GLS 21%, GWI 2335 mmHg%, GCW 2619 mmHg%, GWW 58 mmHg%, GWE 97%). **b** and **d**17-segment bull’s-eye diagram of MW index and myocardial work efficiency from the control one (GLS 22%, GWI 2283 mmHg%, GCW 2743 mmHg%, GWW 46 mmHg%, GWE 98%)

Global myocardial work index (GWI) was the total work derived from the area of LV PSL (Fig. 2). Global constructive myocardial work (GCW), which represented positive work, was performed by contracting myocytes during systole and elongating myocytes during isovolumic relaxation. Global wasted myocardial work (GWW), representing the energy loss, was defined as myocardial lengthening during systole and shortening during isovolumic relaxation [9]. Global myocardial work efficiency (GWE) was the ratio of GCW to sum of GCW and GWW.

### Statistical analysis

All statistical data was processed using standard statistical software SPSS (ver. 24.0, IBM, Chicago, IL). Continuous variables were confirmed for normal distribution by the Kolmogorov-Smirnov test and expressed as mean values±standard deviation (SD). Differences between the two groups in continuous variables were analyzed using independent *t*-test for normal distribution and Mann-Whitney U test for non-normal distribution. Pearson correlation analysis was performed between conventional echocardiography and MW parameters. Receiver operating characteristic curve (ROC) was applied to find optimal parameters to predict LV systolic function in athletes with values of sensitivity, specificity and the area under ROC (AUC). Intra-observer and inter-observer variability were assessed in 10 randomly selected subjects. *P*-value<0.05 were considered as statistically significance.

## Results

### Participant characteristics

Demographic data and conventional echocardiographic parameters of the athletes and controls were shown in Table 1. The values of height, weight, BSA, body mass index (BMI) of the athletes were higher, while those of heart rate (HR) were lower than that of the controls. All of the data above showed statistically significant differences (*P*<0.05). However, there were no statistical differences in age, systolic blood pressure (SBP) and diastolic blood pressure (DBP) between the two groups (*P*>0.05). Compared with the control group, the LVDd, IVSTd, PWTd, RWT, LVM index (LVMI), end-diastolic volume index (EDVI), end-systolic volume index (ESVI), stroke volume index (SVI) and cardiac index (CI) of the athlete group were significantly greater (*P*<0.05), but LVEF was excepted (*P*>0.05).

**Table 1:** Demographic and echocardiographic parameters of the study population

Variable	Athlete group (*n* = 36)	Control group (*n* = 32)	*P*-value
**Age (years)**	19.50 ± 1.38	20.03 ± 1.06	0.083
**Height (m)**	1.78 ± 0.07	1.74 ± 0.04	0.005*
**Weight (kg)**	84.06 ± 14.92	68.34 ± 7.50	<0.001*
**SBP (mmHg)**	127.58 ± 6.97	125.88 ± 8.84	0.377
**DBP (mmHg)**	81.06 ± 4.39	80.53 ± 3.76	0.601
**HR (bpm)**	58 ± 9.08	68 ± 11.07	<0.001*
**BSA (m**^**2**^**)**	2.02 ± 0.19	1.82 ± 0.10	<0.001*
**BMI (kg/m**^**2**^**)**	26.25 ± 3.75	22.43 ± 2.27	<0.001*
**IVSTd (mm)**	10.50 ± 0.48	8.47 ± 0.48	<0.001*
**PWTd (mm)**	10.67 ± 0.51	8.69 ± 0.0.60	<0.001*
**LVDd (mm)**	50.57 ± 1.87	45.51 ± 2.56	<0.001*
**RWT**	0.42 ± 0.01	0.38 ± 0.03	<0.001*
**LVMI (g/m**^**2**^**)**	99.88 ± 11.76	70.02 ± 9.41	<0.001*
**LV-EDVI (mL/m**^**2**^**)**	80.17 ± 11.80	59.64 ± 8.42	<0.001*
**LV-ESVI (mL/m**^**2**^**)**	29.46 ± 5.37	22.15 ± 3.36	<0.001*
**LV-SVI (mL/m**^**2**^**)**	50.87 ± 7.42	37.20 ± 6.28	<0.001*
**LV-CI (1/min/m**^**2**^**)**	3.13 ± 0.67	2.50 ± 0.60	<0.001*
**LV-EF (%)**	62.83 ± 3.19	63.33 ± 2.88	0.907

*SBP* Systolic blood pressure, *DBP* Diastolic blood pressure, *HR* Heart rate, *BSA* Body surface area, *BMI* Body mass index, *IVSTd* Diastolic interventricular septal thickness, *PWTd* Diastolic posterior wall thickness, *LVDd* Left ventricular end-diastolic diameter, *RWT* Relative wall thickness, *LVMI* Left ventricular mass index, *EDVI* End-diastolic volume index, *ESVI* End-systolic volume index, *SVI* Stroke volume index, *CI* Cardiac index, *EF* Ejection fraction

*P < 0.05, indicating significantly different from the control group

### MW and ROC analysis

**Fig. 3:**
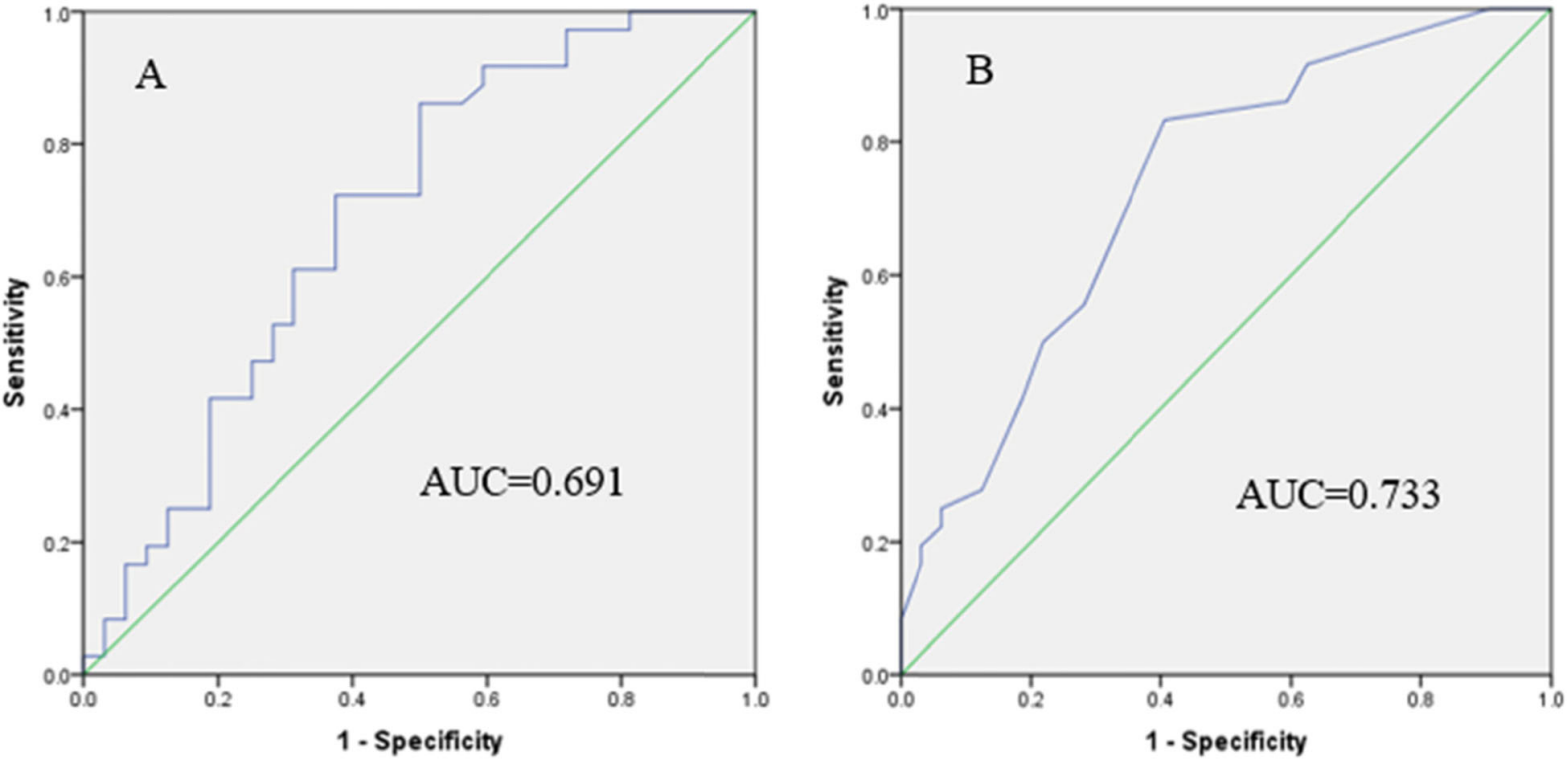
Receiver operating characteristic curve (ROC) for prediction of LV dysfunction in athletes. **a** GWW. **b** GWE. GWE was superior to the other parameters to predict LV performance (AUC = 0.733; 95%CI, 0.613–0.853; *P*<0.05), with the cutoff value of 97.16 mmHg, sensitivity of 83.3%, specificity of 59.4%. GWW was less predictive (AUC = 0.691; 95%CI, 0.562–0.819; *P*<0.05)

The comparison of LV strain and MW parameters between the two groups were summarized in Table 2. Compared with the control group, the values of GWW and PSD increased, while those of GWE decreased in the athlete group, with striking differences (*P*<0.05). Although the level of GWI, GCW and GLS in the athlete group were lower than that of the control group, there were no statistical differences (*P*>0.05). In correlation analysis (Table 3), SBP exhibited a significant positive correlation with MW parameters, and GLS was related to GWI, GCW and GWE. IVST, PWT, LVM, EF and PSD had good correlation with GWW and GWE, respectively, all with statistical significance (*P*<0.05). ROC was used to determine whether MW parameters in athletes were able to forecast the changes of LV systolic function. According to the results of ROC analysis (Table 4, Fig. 3), the optimal cutoff value for GWW was 41.84 mmHg, with sensitivity of 86.1% and specificity of 50%, and the optimal cutoff value for GWE was 97.16%, with sensitivity of 83.3% and specificity of 59.4%. The positive and negative predictive value of GWW were 63.3 and 78%, and GWE were 66.7 and 78%, respectively. Therefore, GWE (AUC = 0.733) was superior to GWW (AUC = 0.691) and other parameters on detecting LV systolic function in athletes.

**Table 2:** Left ventricular myocardial work and strain parameters analysis

Variable	Athlete group (*n* = 36)	Control group (*n* = 32)	*P*-value
GWI (mmHg%)	2030.37 ± 241.52	2050.82 ± 192.71	0.703
GCW (mmHg%)	2336.14 ± 299.20	2384.37 ± 241.58	0.471
GWW (mmHg%)	61.17 ± 29.85	45.33 ± 28.50	0.007*
GWE (%)	96.50 ± 1.35	97.33 ± 1.03	0.001*
GLS (%)	20.71 ± 2.02	21.42 ± 1.49	0.108
PSD (ms)	36.09 ± 7.30	30.35 ± 6.91	0.001*

*GWI* Global myocardial work index, *GCW* Global constructive myocardial work, *GWW* Global wasted myocardial work, *GWE* Global myocardial work efficiency, *GLS* Global longitudinal strain, *PSD* Peak strain dispersion

*P < 0.05, significantly different from the control group

**Table 3:** Correlation analysis of conventional echocardiography and myocardial work parameters

Variable	GWI (mmHg)	GCW (mmHg)	GWW (mmHg)	GWE (%)
IVSTd (mm)	0.098	0.153	0.504*	−0.423*
PWTd (mm)	0.112	0.130	0.373*	−0.304*
LVM (g)	0.102	0.122	0.443*	−0.388*
EF (%)	0.161	0.088	−0.348*	0.354*
SBP (mmHg)	0.581*	0.562*	0.476*	−0.483*
GLS (%)	0.771*	0.806*	−0.104	0.362*
PSD (ms)	0.074	−0.052	0.653*	−0.695*

Values are correlation coefficient (*r*); *LVM* Left ventricular mass

* *P*<0.05 indicated that the correlation was statistically significant

**Table 4:** Receiver operating characteristic curve analysis

Variable	AUC (SE)	AUC (95%CI)	Cutoff value	Sensitivity	Specificity
GWI (mmHg%)	0.566 (0.071)	0.428–0.704	2012.50	55.6%	65.6%
GCW (mmHg%)	0.579 (0.070)	0.441–0.717	2299.34	55.6%	65.6%
GWW (mmHg%)	0.691 (0.065)*	0.562–0.819	41.83	86.1%	50.0%
GWE (%)	0.733 (0.061)*	0.613–0.853	97.16	83.3%	59.4%
GLS (%)	0.627 (0.069)	0.492–0.761	19.85	41.7%	90.6%

*AUC* the area under receiver operating characteristic curve, *SE* Standard error

*P<0.05, significantly different from the control group

### Repeatability and reproducibility

The intra-observer correlation coefficients of GWI, GCW, GWW and GWE were 0.979, 0.976, 0.873 and 0.860, respectively. The inter-observer correlation coefficients of GWI, GCW, GWW and GWE were 0.948, 0.985, 0.957 and 0.878, respectively. It showed good repeatability and reproducibility in MW parameters.

## Discussion

As is known to all that moderate exercise not only benefits to reduce the risk of developing cardiovascular disease but also promotes physical and mental health [16]. Nevertheless, more haste less speed. As repeated professional and systematic training, athletes’ hearts would undergo chambers dilatation, ventricular wall thickening, capacity enlargement, arrhythmia and more seriously, SCD following [17, 18]. Therefore, it seemed clinically significant that every effort would be made to distinguish physiological adaptation from pathological abnormality of athletes’ hearts as soon as possible in case of putting the athletes at risk [4, 17, 19]. Heart changes depended on the type of exercises [20]. In this study, structural and capacity parameters of LV were increased in the athlete group with RWT (0.42 ± 0.01, *P*<0.001) exceeding the reference value (RWT >0.42) and LVMI (99.88 ± 11.76, *P*<0.001) remaining within standard range. All of these suggested that concentric remodeling of LV in athletes was displayed after recurrent exercise, which was well consistent with previous researches [6, 17].

After prolonged exercise, the augment of RWT and the dropping of capillary density in strength athletes would lead to increased regional myocardial oxygen consumption and insufficient blood supply to myocardium [16, 21]. Moreover, given the complicated three-layer structure of myocardium, microvascular dysfunction and myocardial fibrosis were more likely to occur in the endocardium due to low compliance and high oxygen expenditure of the vascular which led to limited ability to dilate blood vessels and store blood [22, 23]. The description of these changes has led to a more comprehensive understanding of reduced longitudinal strain of LV, as well as the decrease of GWI and GCW that could be supposed to reflect LV performance and mechanics globally [11]. In this study, compared with the control group, the reduction in GWI and GCW in the athlete group was not statistically significant, but suggested that the myocardial metabolism was decreased and LV systolic function was impaired to some extent. LV remodeling can lead to abnormal electrophysiology of the myocardium, resulting in conduction disorders of myocardial cells, and reducing the effectiveness of coordinated contraction of the myocardium. Significantly, increased PSD in the athlete group draw attention to the points that the systolic synchronization of LV had been destroyed and nonuniformity of ventricular wall motion would reduce the mechanical efficiency during cardiac ejection [14]. In addition, the strength athletes were characterized by high LV afterload which would lead to the increased of LV stiffness. Eventually, result in more energy loss expressed as increased GWW and reduced GWE.

Previous studies have demonstrated that the reduction of GLS in athletes was an early sign of LV dysfunction [6]. However, in this study, compared with the control group, the athlete group shew increased GWW and reduced GWE, while EF and GLS having no statistical differences. The above results illustrated that subclinical changes in LV systolic function have occurred in athletes and MW indexes were more sensitive to provide early diagnosis value for LV performance among athletes [24]. According to the ROC analysis about LVMW parameters, GWE (AUC = 0.733) was considered of being the best predictor of LV systolic function in athletes and superior to GLS (AUC = 0.627) which was principally limited by the defect of being load-dependent and might lead to errors in judging LV performance [25].

### Limitations

Several limitations to this study need to be acknowledged as follows: (i) The sample scale was small. The research had only considered the context of young male strength athletes, regardless of the female and endurance ones. (ii) The use of brachial cuff pressure to evaluate LVMW might not be applicable to all the situations such as aortic stenosis, which was likely to impact the accuracy of LVMW parameters evaluation [7, 9]. However, the validity of the non-invasive approach to evaluate LVMW has been confirmed by previous studies in different hemodynamic conditions [26]. (iii) In addition, 2D-STE analysis was limited by high image quality to obtain exact strain values [18].

## Conclusions

In summary, this study revealed that the athletes after long-term intensive training not only generated LV concentric remodeling, but also developed subtle subclinical changes in LV contractility performed by increased GWW and decreased GWE. Compared with the conventional echocardiographic indicators, the MW derived from novel non-invasive PSL was a promising tool to evaluate progressive changes of LV performance and produce incremental practical significance to detect myocardial function abnormalities of LV in order to prevent athletes from being SCD in the early stage.

## Data Availability

All data generated or analyzed during this study are included in this published article.

## Abbreviations

LV: Left ventricular
PSL: Pressure-strain loop
MW: Myocardial work
2D-STI: Two-dimensional speckle tracking imaging
GWI: Global myocardial work index
GCW: Global constructive myocardial work
GWW: Global wasted myocardial work
GWE: Global myocardial work efficiency
GLS: Global longitudinal strain
PSD: Peak strain dispersion
SCD: Sudden cardiac death
RWT: Relative wall thickness
EF: Ejection fraction
